# Biogenic silver nanoparticles as antifungal agents

**DOI:** 10.3389/fchem.2022.1023542

**Published:** 2022-10-06

**Authors:** Javier Mussin, Gustavo Giusiano

**Affiliations:** Mycology Department, Consejo Nacional de Investigaciones Científicas y Tecnológicas (CONICET), Instituto de Medicina Regional, Universidad Nacional del Nordeste (UNNE), Resistencia, Argentina

**Keywords:** silver nanoparticles, antimicrobial, antifungal, mycoses, green synthesis, biogenic synthesis, mechanism of action, resistance mechanism

## Abstract

In recent years, an increase in multidrug-resistant fungal strains has been observed, which, together with the limited number of clinically available antifungal agents, highlights the need for the development of new antifungal agents. Due to the proven antifungal activity of silver nanoparticles (AgNPs), there is a growing interest in their use in the treatment of fungal infections. Nanoparticles are usually synthesised through a variety of physical and chemical processes that are costly and pollute the environment. For this reason, biogenic synthesis is emerging as an environmentally friendly technology and new strategies are increasingly based on the use of biogenic AgNPs as antifungal agents for clinical use. The aim of this review is to compare the antifungal activity of different biogenic AgNPs and to summarise the current knowledge on the mechanisms of action and resistance of fungi to AgNPs. Finally, a general analysis of the toxicity of biogenic AgNPs in human and veterinary medicine is performed.

## Introduction

In recent decades, fungal infections have increased and become a major public health threat. More than 300 million people suffer from severe fungal diseases and more than 2 million people die each year from mycoses, making fungal diseases one of the leading causes of death worldwide. Moreover, the problem of mycoses is exacerbated by the increase in emerging pathogenic fungi, but also by resistance to the limited antifungal drugs available, which significantly reduces the effectiveness of treatments (Denning et al., 2017; GAFFI, 2021).

From this perspective, *Candida auris* infections have become a global threat to human health for four main reasons: It has caused public health outbreaks; it is difficult to identify using standard laboratory methods; it has a high mortality rate; and some strains are resistant to all classes of available antifungal drugs commonly used to treat *Candida* infections (Du et al., 2020; CDC, 2021). On the other hand, there are certain mycoses whose treatment remains ineffective and leads to disability, including certain superficial fungal infections and implantation mycoses. This situation, which has a significant social impact, also includes the economic factor caused by the need for expensive drugs and lengthy medical care, which in many cases leads to patients not adhering to treatment (GAFFI, 2022).

The lack of antifungal drugs is mainly due to the difficulty in finding selective therapeutic targets against fungi, as they have a cellular and molecular biology very similar to that of animal cells (Konopka et al., 2019). This has prompted research into metals as alternative antifungal agents or as cofactors/adjuvants that enhance the efficacy of existing drugs (Xu et al., 2013; Mussin et al., 2019; Mani Chandrika and Sharma, 2020; Cruz-Luna et al., 2021; Lin et al., 2021).

Among the various metals, silver has a long history in medicine as an antimicrobial agent (Rai et al., 2009). Currently, silver nanoparticles (AgNPs) occupy a prominent place as potential antifungal agents for clinical use due to their broad spectrum of antimicrobial activity and their enormous number of applications in the health sciences, ranging from topical formulations to catheters impregnated with AgNPs (Rai et al., 2009; Ahamed et al., 2010; Burdușel el et al., 2018; Mosleh-Shirazi et al., 2021a).

AgNPs are particles that have all three dimensions on the nanometre scale (10^–9^ m). These nanoparticles can be synthesised by physical, chemical and biological methods. However, biological synthesis stands out because it is environmentally friendly, economically viable and easy to transfer to industrial production (Siddiqi et al., 2018; Mussin et al., 2021).

Biogenic synthesis of AgNP is a green synthesis method that uses organisms (plants, bacteria, fungi and algae) as a source of biomolecules that serve as reducing agents for silver ions (Ag^+^), coating agents and stabilisers of AgNP (Siddiqi et al., 2018; Rozhin et al., 2021).

Since natural coating agents can impart some functionality to the nanoparticles, such as antioxidant properties, anti-inflammatory properties, lower toxicity, modulation of immune response, etc., they are considered better candidates for use in human and veterinary medicine compared to nanoparticles obtained by physical and chemical methods (Vigneshwaran et al., 2007; Haggag et al., 2019; Sadeghipour et al., 2020; Mosleh-Shirazi et al., 2021b; Mujaddidi et al., 2021; Mussin et al., 2021; Rozhin et al., 2021). However, there are no organised data on the antifungal activity of biogenic AgNPs, nor on the mechanisms of action and resistance of fungi to AgNPs. For this reason, this review aims to compare the antifungal activity of different biogenic AgNPs and to summarise the current knowledge on the mechanisms of action and resistance of fungi to AgNPs. Finally, a general analysis of the toxicity of biogenic AgNPs in human and veterinary medicine is performed.

## Antifungal activity of biogenic AgNPs

Thanks to their broad spectrum of antimicrobial activity and their ability to effectively inhibit biofilm formation, biogenic AgNPs have become one of the most promising options to reduce morbidity and mortality associated with fungal infections caused by resistant fungi (Ahamad et al., 2022).

The antimicrobial activity (antifungal, antibacterial, antiviral, etc.) of biogenic AgNPs is largely determined by the following factors:

### The organism used for synthesis

Living organisms produce a variety of biological molecules (metabolites, proteins, lipids, etc.) that are involved in the reduction of Ag^+^, but also stabilise AgNPs and prevent their agglomeration. Moreover, biomolecules are the main protagonists of biological synthesis, as they determine the coating agents that can confer the desired biological properties to the synthesised AgNPs, such as: antioxidant activity, stronger antimicrobial activity, lower toxicity towards human/animal cells, modulation of the immune response and much more (Mussin et al., 2021; Spagnoletti et al., 2021).

It has even been shown that different AgNPs can be obtained from different strains of the same species under identical synthesis conditions (El-Bendary et al., 2021). Therefore, the correct identification of the species used and their preservation in a culture collection or herbarium is crucial.

### The growth conditions of organism

The chemical composition of the same organism may vary according to the conditions of growth. It is therefore important that these conditions are well defined, especially if you are aiming for industrial production. In the case of plants, several points also need to be defined, such as the part of the plant to be used, the time of harvesting, post-harvest treatment, etc., (Mussin and Giusiano, 2020). All these aspects can influence the chemical composition and thus the properties of the synthesised AgNPs.

### The physicochemical properties of AgNPs

The size, shape and coating agent of biogenic AgNPs determine their antifungal activity. These properties are strongly influenced by the synthesis conditions, such as: Temperature, reaction time, pH, biological molecules, molar ratio of reagents, speed and type of stirring, etc. (Song and Kim, 2009; El Badawy et al., 2010; Monteiro et al., 2012; Li et al., 2013; Agnihotri et al., 2014; Gavade et al., 2015; Ahmed et al., 2016). Therefore, standardisation of the synthesis conditions allows obtaining identical nanoparticles in each production batch.

### Target organism

Due to genetic variability between species of the same genus and between strains of the same species, a given nanoparticle may exhibit different levels of antimicrobial activity (Mussin et al., 2019, 2021). Therefore, to obtain meaningful statistical values, the test must be performed against a considerable number of strains of the same species.

In addition, when assessing antimicrobial activity, it is important to note that a standardised method should be used that is widely accepted by the scientific community, as parameters such as the concentration of the inoculum, temperature and incubation time, among others, influence the assessment of antimicrobial activity. One of the most widely accepted methods is the broth microdilution method proposed by the Clinical and Laboratory Standards Institute (CLSI). This method provides a quantitative assessment of the *in vitro* inhibitory activity of a compound against a microorganism by determining the minimum inhibitory concentration (MIC), defined as the lowest concentration of the compound that can inhibit the growth of the microorganism, expressed in μg/ml. In addition, the method provides for the use of reference strains, quality control strains and positive inhibitory controls (antimicrobial drugs for clinical use) to ensure that the microdilution test is performed correctly and that the results are reproducible and comparable.

Table 1 summarises the most important papers in which the MIC of biogenic AgNPs against fungi of clinical importance was determined using a broth dilution method. Analysis of these studies leads us to the following conclusions:

• Fungi and plants are the main organisms that have been used for the synthesis of biogenic AgNPs to study antifungal activity.
• Comparing the works in which the same species was used to synthesise AgNPs (Marcato et al., 2012; Ishida et al., 2014; Longhi et al., 2016; Fonseca et al., 2022), we found that nanoparticles with different properties can be obtained from the same species. Therefore, it is important to consider all the above aspects about the factors that determine the antifungal activity of a biogenic AgNP, otherwise each synthesised nanoparticle must be considered as a different compound.
• Candida albicans ATCC 90028 is the most commonly used reference strain for evaluating the antifungal activity of biogenic AgNPs.
• Fluconazole and itraconazole are the main control drugs used.
• Biogenic AgNPs show a MIC range of 0.002–315.5 μg/ml against different fungal species. However, considering only the papers in which a control drug and a quality control strain were used for the CLSI broth microdilution method (*Candida krusei* ATCC 6258 and *Candida parapsilosis* ATCC 22019), the MIC range of AgNPs was 0.03–4 μg/ml against the tested fungal species. This shows, firstly, the importance of using standardised method controls and, secondly, that biogenic AgNPs have similar or even better antifungal activity than certain clinically used antifungal agents.
• Antifungal activity varies depending on the biogenic AgNP synthesised. Against the quality control strains, *Candida krusei* ATCC 6258 and *Candida parapsilosis* ATCC 22019, the biogenic AgNPs showed MIC ranges of 0.125–4 μg/ml and 0.125–6.25 μg/ml, respectively.

**TABLE 1:** Antifungal activity of biogenic AgNPs.

Synthesis from	Size (nm)	Target (number of strains)	MIC (µg/ml)	Control drug					Ref
				FLZ	ITZ	TER	NYT	AMP	
Plants									
***Acanthospermum australe* (Loef.) Kuntze**	12–16	*Candida albicans* ATCC 90028	4		0.125				Mussin et al. (2021)
		*Candida albicans* (24)	4^a^		≤0.125				
		*Candida glabrata* ATCC 2001	4		0.5				
		*Candida glabrata* (6)	4^a^		0.03				
		*Candida krusei* ATCC 6258	4		0.5				
		*Candida krusei* (21)	4^a^		≤0.5				
		*Candida tropicalis* ATCC 750	4		0.125				
		*Candida tropicalis* (16)	4^a^		≤0.125				
		*Candida parapsilosis* (11)	8^a^		≤0.125				
		*Epidermophyton foccosum* (4)	4^a^		≤0.03				
		*Malassezia globosa* CBS 7986	0.125		0.015				
		*Malassezia globosa* (9)	0.125^a^		0.03				
		*Malassezia furfur* CBS 7019	0.25		0.03				
		*Malassezia furfur* (40)	0.25^a^		0.03				
		*Malassezia restricta*	1		0.03				
		*Malassezia sympodialis* CBS 7222	0.03		0.03				
		*Malassezia sympodialis* (25)	0.03^a^		0.03				
		*Microsporum canis* (33)	4^a^		≤0.125				
		*Microsporum gypseum* (19)	16^a^		≤0.25				
		*Trichophyton mentagrophytes* (22)	16^a^		≤1				
		*Trichophyton rubrum* (31)	4^a^		≤0.5				
		*Trichophyton tonsurans* (6)	4^a^		≤0.125				
***Allium cepa***	1–9	*Fusarium avenaceum*	110						Gautam et al. (2020)
		*Fusarium culmorum*	110						
		*Fusarium graminearum*	90						
***Allium sativum***	2–10	*Fusarium avenaceum*	90						Gautam et al. (2020)
		*Fusarium culmorum*	110						
		*Fusarium graminearum*	110						
***Annona reticulata***	7.67–8.34	*Candida albicans*	62.5						Parthiban et al. (2019)
***Caesalpinia ferrea* (Tul.) Martius**	30–50	*Candida albicans* ATCC 10231	312.5				0.331	0.125	Soares et al. (2018)
		*Candida glabrata* CCT 0728	1,250				0.663	0.25	
		*Candida guilliermondii* CCT 1890	156.25				1.326	0.0312	
		*Candida kruzei* CCT 1517	312.5				2.64	2	
***Citrus limetta***	18	*Candida albicans* MTCC 3018	6.69						Dutta et al. (2020)
		*Candida glabrata* MCC 1445	10.7						
		*Candida parapsilosis* MCC 1438	10.7						
		*Candida tropicalis* MCC 1434	10.7						
***Elettaria cardamomum***	5–80	*Aspergillus niger* ITCC 7122	8						Jamdagni et al. (2021)
		*Alternaria alternata* ITCC 6531	32						
		*Botrytis cinerea* ITCC 6192	32						
		*Fusarium oxysporum* ITCC 55	32						
		*Penicillium expansum* ITCC 6755	64						
***Eucalyptus camaldulensis***	8.65	*Candida albicans* ATCC 90028	0.25					0.25	Wunnoo et al. (2021)
		*Candida albicans* (20)	0.25–0.5					0.25–0.5	
***Lysiloma acapulcensis***	1.2–62	*Candida albicans* ATCC 49476	0.13						Garibo et al. (2020)
***Mangifera indica***	65	*Candida albicans* PTCC 5027	0.016	0.002					Salati et al. (2018)
		*Candida albicans* (11)	0.016^a^	0.008					
		*Candida glabrata* PTCC 5297	0.002	0.004					
		*Candida glabrata*	0.016	0.004					
		*Candida krusei* PTCC 5295	0.016	0.004					
		*Candida krusei* (7)	0.016^a^	0.002					
***Maytenus royleanus***	10–15	*Candida albicans*	125						Ahmad et al. (2016)
		*Candida tropicalis*	62.5						
***Pulicaria vulgaris* Gaertn**	14.3–50.7	*Candida albicans* ATCC 10231	60						Sharifi-Rad and Pohl, (2020)
		*Candida glabrata* ATCC 90030	40						
***Rosa canina***	13–21	*Candida albicans*	128						Gulbagca et al. (2019)
***Tropaeolum majus***	ND	*Aspergillus niger*	125						Valsalam et al. (2019)
		*Candida albicans*	250						
		*Mucor* sp	31,2						
		*Penicillium notatum*	31,2						
		*Trichoderma viridiae*	62,5						
***Zingiber officinale***	1–6	*Fusarium avenaceum*	110						Gautam et al. (2020)
		*Fusarium culmorum*	90						
		*Fusarium graminearum*	110						
Bacteria									
***Anabaena variabilis***	11–15	*Candida albicans* MCC 1151	12.5						Ahamad et al. (2022)
***Citrobacter* sp**	5–15	*Candida albicans*	100	16					Mondal et al. (2020)
		*Candida glabrata*	150	16					
		*Candida tropicalis*	150	˃64					
***Lysinibacillus sphaericus***	3–38	*Candida albicans* ATCC 10231	76–228						El-Bendary et al. (2021)
		*Aspergillus niger*	305						
***Nostoc linckia***	5–60	*Candida albicans* MTCC 4748	0.61^b^						Vanlalveni et al. (2018)
		*Aspergillus niger*	0.40^b^						
***Pseudomonas indica***	2.4–53.5	*Mucor racemosus*	100						Salem et al. (2022)
		*Rhizopus microsporus*	50						
		*Syncephalastrum racemosum*	50						
Fungi									
***Arthroderma fulvum***	13–18	*Aspergillus flavus* IFM 55648	2	>64	0.125				Xue et al. (2016)
		*Aspergillus fumigatus* IFM 40808	1	>64	0.030				
		*Aspergillus terrrus* JLCC 30844	1	>64	0.250				
		*Candida albicans* ATCC 90028	0.5	0.250	0.030				
		*Candida krusei* ATCC 6258	0.125	16	0.250				
		*Candida parapsilosis* ATCC 22019	0.125	8	0.250				
		*Candida tropicalis* JLCC 31384	0.250	0.250	0.250				
		*Fusarium moniliforme* JLCC 31463	4	>64	>16				
		*Fusarium oxysporum* JLCC 31768	2	>64	>16				
		*Fusarium solani* JLCC 30866	2	>64	>16				
***Aspergillus oryzae***	19–60	*Trichophyton rubrum* ATCC MYA-443	>7.5	≤16	≤0.126	4			Pereira et al. (2014)
		*Trichophyton rubrum* (8)	>7.5	≤32	≤1	≤0.25			
***Aspergillus sydowii***	1–24	*Aspergillus flavus* IFM 55648	1	>64	0.125				Wang et al. (2021)
		*Aspergillus fumigatus* IFM 40808	2	>64	0.03				
		*Aspergillus terrus* JLCC 30844	2	>64	0.25				
		*Candida albicans* ATCC 90028	0.25	0.25	0.03				
		*Candida glabrata* ATCC 66032	0.125	8	0.25				
		*Candida parapsilosis* ATCC 22019	0.25	8	0.25				
		*Candida tropicalis* ATCC 9928	0.125	0.25	0.25				
		*Cryptococcus neoformans* ATCC 36556	0.25	2	0.0625				
		*Fusarium moniliforme* JLCC 31463	2	>64	>16				
		*Fusarium oxysporum* JLCC 30866	4	>64	>16				
		*Fusarium solani* ATCC 36031	1	>64	>16				
		*Sporothrix schenckii* JLCC 32757	0.25	>64	0.125				
***Aspergillus terreus***	7–23	*Aspergillus niger* ATCC 16404	0.312	0.156					Lotfy et al. (2021)
		*Candida albicans* ATCC 10231	1.25	0.156					
***Candida glabrata***	2–15	*Candida albicans*	62						Jalal et al. (2018)
		*Candida dubliniensis*	125						
		*Candida glabrata*	250						
		*Candida krusei*	125						
		*Candida parapsilosis*	250						
		*Candida tropicalis*	250						
***Cryptococcus laurentii***	35 (74%) and 400 (13%)	*Alternaria* sp. NRRL 6410	4						Fernández et al. (2016)
		*Aspergillus niger* NRRL 1419	4						
		*Botrytis cinerea* BNM 0528	4						
		*Penicillium expansum*	4						
		*Rhizopus* sp. NRRL 695	4						
***Epicoccum nigrum***	1–22	*Aspergillus flavus* IFM 55648	0.5	>64	0.125				Qian et al. (2013)
		*Aspergillus fumigatus* IFM 40808	1	>64	0.03				
		*Candida albicans* ATCC 90028	0.5	0.25	0.03				
		*Candida krusei* ATCC 6258	0.125	16	0.25				
		*Candida parapsilosis* ATCC 22019	0.125	8	0.25				
		*Candida tropicalis* JLCC 31384	1	0.25	0.03				
		*Cryptococcus neoformans* IFM 45687	0.25	2	0.0625				
		*Fusarium solani* JLCC 30866	1	>64	>16				
		*Sporothrix schenckii* JLCC 32757	0.25	>64	0.125				
***Fusarium oxysporum***	ND	*Candida albicans* ATCC 26790	4.35	>128					Longhi et al. (2016)
		*Candida albicans* (2)	1.74	64–128					
***Fusarium oxysporum***	2–100	*Trichophyton rubrum*	1–2					1–5	Marcato et al. (2012)
***Fusarium oxysporum***	4.8–64.9	*Candida albicans* ATCC 10231	1.68						Ishida et al. (2014)
		*Candida albicans* ATCC 24433	1.68						
		*Candida glabrata* ATCC 2001	1.68						
		*Candida krusei* ATCC 6258	0.84						
		*Candida parapsilosis* ATCC 22019	0.84						
		*Candida tropicalis* ATCC 13803	1.68						
		*Cryptococcus gattii* ATCC 56990	0.84						
		*Cryptococcus neoformans* ATCC 28957	0.42						
***Fusarium oxysporum***	14.9–41.1	*Candida albicans* (16)	7.8^a^	1		0.03125	2	0.5	Fonseca et al. (2022)
		*Candida dubliniensis*	15.6	32		0.03125	2	2	
		*Candida glabrata*	7.8	>64		>16	2	0.5	
		*Candida parapsilosis*	7.8	16		0.125	8	2	
		*Candida tropicalis* (6)	7.8^a^	2		0.03125	4	2	
***Metarhizium roberts***	15–25	*Candida albicans* ATCC 10231	1.56						Różalska et al. (2018)
		*Candida albicans* ATCC 90028	6.25	1					
		*Candida glabrata* ATCC 90030	3.12						
		*Candida parapsilosis* ATCC 22019	6.25						
***Penicillium chrysogenum***	19–60	*Trichophyton rubrum* ATCC MYA-443	0.5	≤16	≤0.126	4			Pereira et al. (2014)
		*Trichophyton rubrum* (8)	0.5–5	≤32	≤1	≤0.25			
***Pleurotus ostreatus***	4–15	*Candida albicans* (3)	5–7	13–19			8		Yehia and Al-Sheikh, (2014)
		*Candida glabrata* (4)	16	23–33			7		
		*Candida krusei* (2)	4–11	13–19			5–7		
		*Candida parapsilosis*	10	16			5–7		
		*Candida tropicalis* (2)	7–28	16			5		
***Rhodotorula glutinis***	15 (65%), 160 (17%) and 220 (18%)	*Alternaria* sp. NRRL 6410	2						Fernández et al. (2016)
		*Aspergillus niger* NRRL 1419	2						
		*Botrytis cinerea* BNM 0528	2						
		*Penicillium expansum*	2						
		*Rhizopus* sp. NRRL 695	2						

a The most frequent MIC, is expressed.

b MIC, expressed as nM; ATCC, american type culture collection; BNM, national bank of microorganisms; CBS, westerdijk fungal biodiversity institute, formerly known as “Centraal Bureau voor Schimmelcultures"; CCT: collection of tropical cultures.

FLZ, fluconazole; ITZ: itraconazole; TER, terbinafine; NYT, nystatin; AMP, Amphotericin B; IFM, institute for food microbiology; ITCC, indian type culture collection; JLCC, culture collection of jilin university; MCC, microbial culture collection from the national centre for microbial resource; MIC, minimum inhibitory concentration; MTCC, microbial type culture collection and gene bank; ND, no determined; NRRL, agricultural research service culture collection; PTCC, persian type culture collection.

## Mechanism of action

**FIGURE 1:**
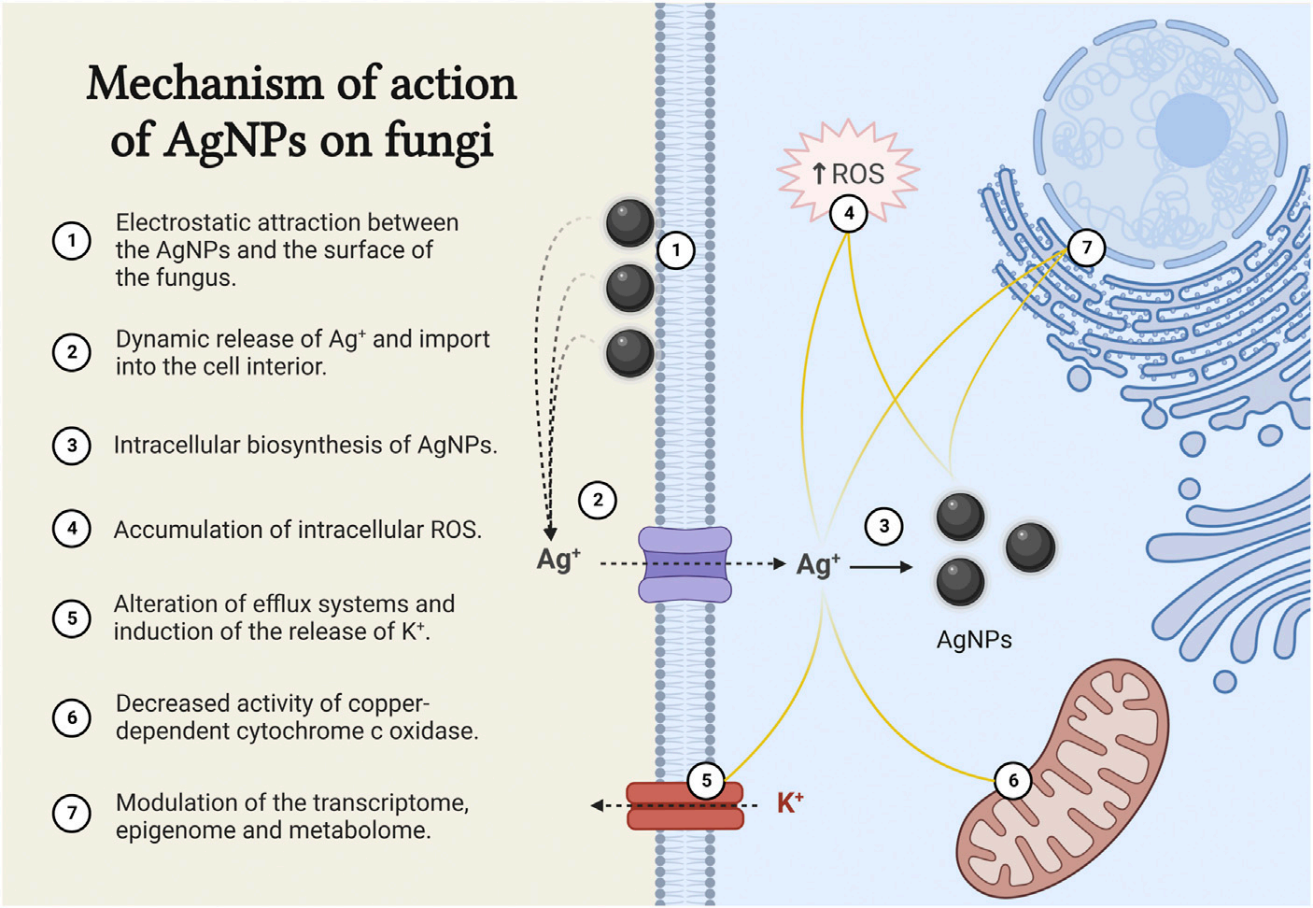
Mechanism of action of AgNPs on fungi. The figure was created with BioRender.com.

As mentioned earlier, the antifungal activity of biogenic AgNPs is highly dependent on the size, shape and coating agents. The great diversity of biogenic AgNPs therefore makes it difficult to decipher a single mechanism of action. For this reason, most research has focused on determining the mechanism of action of chemically synthesised AgNPs, which is attributed to the attachment of AgNPs to the surface of the fungus as a result of electrostatic attraction (Figure 1). The extracellular accumulation of AgNPs leads to a dynamic release of Ag^+^, which actively enter the cell and lead to an increase in the intracellular concentration and also the intracellular biosynthesis of AgNPs (Chwalibog et al., 2010; Le et al., 2012; Vazquez-Muñoz et al., 2014; Lara et al., 2015; Mussin et al., 2019). So far, no cell receptors or membrane channels have been described for the uptake of silver. However, the high-affinity copper transporter (Ctr1) has been identified as an importer of Ag^+^ (Ruta et al., 2018; Horstmann et al., 2019).

### Once inside the cell, Ag^+^ and AgNPs act at different levels

• They lead to an accumulation of intracellular reactive oxygen species (ROS), which trigger apoptosis (Madeo et al., 1999; Hwang et al., 2012; Radhakrishnan et al., 2018).
• The intracellular accumulation of Ag^+^ alters efflux systems. It induces efflux of potassium ions and causes almost complete loss of intracellular potassium ions, resulting in inhibition of plasma membrane H^+^-ATPase activity (Vagabov et al., 2008; Cyert and Philpott, 2013).
• Ag^+^ enter the mitochondria via the mitochondrial copper transporter Pic2, which has a higher affinity for Ag^+^ than for copper ions (Cu^+^). This leads to a decrease in Cu^+^ concentration and an accumulation of Ag^+^ in the mitochondrial matrix, resulting in a decrease in the activity of the copper-dependent cytochrome c oxidase and consequently decreasing the rate of cellular respiration (Vest et al., 2013).
• Ag^+^ and AgNPs modulate the transcriptome, epigenome and metabolome and significantly alter the vital functions of fungal cells. Down-regulation of tricarboxylic acid cycle genes, genes related to redox metabolism and genes involved in ergosterol synthesis and lipid metabolism have been reported, leading to structural changes mainly at the level of biological membranes (Das and Ahmed, 2012; Babele et al., 2019; Horstmann et al., 2019; Barros et al., 2021).

Our studies suggest that AgNPs have fungicidal action against the major fungi that cause skin infections. A fungicidal agent causes death of fungal cells, while a fungistatic agent inhibits the growth or multiplication of the fungus without causing death (Mussin et al., 2021). However, the results are suggestive and further studies should be conducted.

AgNPs have also been shown to be more effective when combined with antifungal drugs. Synergistic effects have been reported with fluconazole, itraconazole, ketoconazole, clotrimazole, terbinafine, natamycin, nystatin, amphotericin B and echinocandins (Gajbhiye et al., 2009; Xu et al., 2013; Padalia et al., 2015; Patra and Baek, 2017; Mussin et al., 2019; Aabed and Mohammed, 2021; Yassin et al., 2022).

## Resistance mechanisms

Since the antifungal activity of AgNPs is the result of several simultaneous processes, this has led to the assumption that fungi cannot develop resistance mechanisms to AgNPs.

Few studies have analysed the possible mechanisms of fungal resistance to silver. Terzioğlu et al. (2020) used the yeast *Saccharomyces cerevisiae* as a model fungal organism to investigate possible molecular mechanisms associated with resistance to silver. Their results suggest that genes involved in cell wall/membrane integrity, endocytosis and vesicular transport activities, oxidative metabolism, cellular respiration and copper homeostasis may play a role in silver resistance. In particular, the missense mutation in the RLM1 gene, which encodes a transcription factor involved in maintaining cell wall integrity and has 707 potential gene targets, may play a key role.

On the other hand, using the filamentous fungus *Aspergillus nidulans*, Antsotegi-Uskola et al. have suggested that the copper-transporting ATPase type PI, CrpA, may play an important role in the development of silver resistance (Antsotegi-Uskola et al., 2017).

Due to the increasing use of silver and AgNPs in many areas of human and veterinary medicine, further research is needed.

## Toxicity

The toxicity of AgNPs depends on the size, shape and coating agents. For biogenic AgNPs, the coating agents play a very important role in terms of toxicity to human cells and modulation of the immune response (Mussin et al., 2021). There is evidence that biogenic AgNPs are more biocompatible than chemically synthesised AgNPs (Khan et al., 2019; Quinteros et al., 2019). However, due to the complex interactions between the different coating agents and eukaryotic cells, each biogenic AgNP should be evaluated individually to confirm its safety in humans and other animals.

The route of administration, exposure time and pharmacokinetics also influence toxicity (Stensberg et al., 2011; Aboelmaati et al., 2021; Mosleh-Shirazi et al., 2021a). Therefore, it remains to be investigated whether the gradual release of Ag^+^ and the broad spectrum of antimicrobial activity of AgNPs may lead to changes in the normal microbiota of humans and animals and whether this may have adverse effects over time.

Another interesting aspect of biogenic AgNPs is the reported synergistic effects with antifungals (Gajbhiye et al., 2009; Xu et al., 2013; Padalia et al., 2015; Patra and Baek, 2017; Mussin et al., 2019; Aabed and Mohammed, 2021; Yassin et al., 2022), suggesting that combined use may reduce toxicity by reducing the required dose of one or both agents.

## Conclusion and future perspective

The increase in multidrug-resistant fungal pathogens and the limited number of clinically available antifungal drugs highlight the need to develop new antifungal strategies to address these problems in the face of an already complicated future.

AgNPs have been presented as a promising solution, but biological AgNPs have been shown to have several advantages over AgNPs produced by chemical and physical methods.

The antifungal activity of the different biogenic nanoparticles varies according to their physicochemical properties, which are determined by the organism used for synthesis, the growth conditions of the organism, the physicochemical properties of the AgNPs and the target organism. An important challenge for future research is therefore to standardise these conditions and determine the key biocomponents involved in the synthesis of AgNPs to produce safe and effective drugs for the treatment of fungal infections.

The wide variety of methods used to evaluate the antifungal activity of these biogenic nanoparticles highlights the need to use internationally accepted methods with appropriate controls to obtain reproducible and comparable results. Since there may be genetic variability within a species, it is important to test a considerable number of isolates of the same species to obtain meaningful results on the antifungal activity of a new agent against a particular species.

Great progress has been made in elucidating the mechanism of action of AgNPs on fungi. They have shown that they can act on multiple targets, which makes them very promising as antifungal agents for clinical use. In addition, further research is being conducted for use in healthcare settings. In the near future, these efforts will lead to a clearer picture of the antifungal potential of biogenic AgNPs and help establish them in the field of veterinary and human mycology.

The broad spectrum of antimicrobial activity and the potential synergistic effects with antifungal drugs make biogenic AgNPs viable alternatives to overcome the problematic infections caused by resistant fungi and the toxicity of currently available drugs. We anticipate that biogenic AgNPs will be used as cost-effective broad-spectrum antifungal agents. However, since toxicity and *in vivo* effects have not yet been sufficiently researched, we think it more likely that they will initially be used in human and veterinary medicine as antimycotics for topical application or as disinfectants for catheters, surgical materials, etc.
